# Sedentary behavior is associated with poor sleep quality during the COVID-19 pandemic, and physical activity mitigates its adverse effects

**DOI:** 10.1186/s12889-023-16041-8

**Published:** 2023-06-12

**Authors:** Luiz Antônio Alves de Menezes-Júnior, Samara Silva de Moura, Amanda Gonçalves Miranda, Amanda Cristina de Souza Andrade, George Luiz Lins Machado-Coelho, Adriana Lúcia Meireles

**Affiliations:** 1 Postgraduate Program in Health and Nutrition, School of Nutrition, Federal University of Ouro, Preto, Ouro Preto, Minas Gerais Brazil; 2 Research and Study Group on Nutrition and Public Health (GPENSC), Federal University of Ouro Preto, Ouro Preto, Brazil; 3 Department of Public Health, Federal University of Mato Grosso, Cuiabá, Mato Grosso Brazil; 4 School of Medicine, Federal University of Ouro Preto, Ouro Preto, Minas Gerais Brazil; 5 Department of Clinical and Social Nutrition, School of Nutrition, Federal University of Ouro, Preto, Ouro Preto, Minas Gerais Brazil

**Keywords:** Sleep disorders, SARS-CoV-2, Sedentary lifestyle, Physical activity, Public health

## Abstract

**Background:**

We aimed to evaluate the association of sedentary behavior (SB) and moderate to vigorous leisure-time physical activity (MVPA) with sleep quality during the COVID-19 pandemic.

**Methods:**

Cross-sectional, population-based study in adults, conducted from October to December 2020 in the Iron Quadrangle region, Brazil. The outcome was sleep quality, evaluated with the Pittsburgh Sleep Quality Index. SB was assessed by self-report of total sitting time, before and during the pandemic. Individuals with ≥ 9 h of total sitting time were classified as SB. In addition, the ratio of time spent in MVPA to time in SB was analyzed. A contrasted directed acyclic graph (DAG) model was constructed to adjust logistic regression models.

**Results:**

A total of 1629 individuals were evaluated, the prevalence of SB before the pandemic was 11.3% (95%CI: 8.6–14.8), and during the pandemic 15.2% (95%CI: 12.1–18.9). In multivariate analysis, the chance of poor sleep quality was 77% higher in subjects with SB ≥ 9 h per day (OR: 1.77; 95% CI: 1.02–2.97). Furthermore, a one-hour increase in SB during the pandemic, increased the chance of poor sleep quality by 8% (OR: 1.08; 95%CI 1.01–1.15). In the analysis of the ratio of MVPA per SB in individuals with SB ≥ 9 h, practicing 1 min of MVPA per hour of SB reduces the chance of poor sleep quality by 19% (OR: 0.84; 95%CI: 0.73–0.98).

**Conclusion:**

SB during the pandemic was a factor associated with poor sleep quality, and the practice of MVPA can reduce the effects of SB.

**Graphical Abstract:**

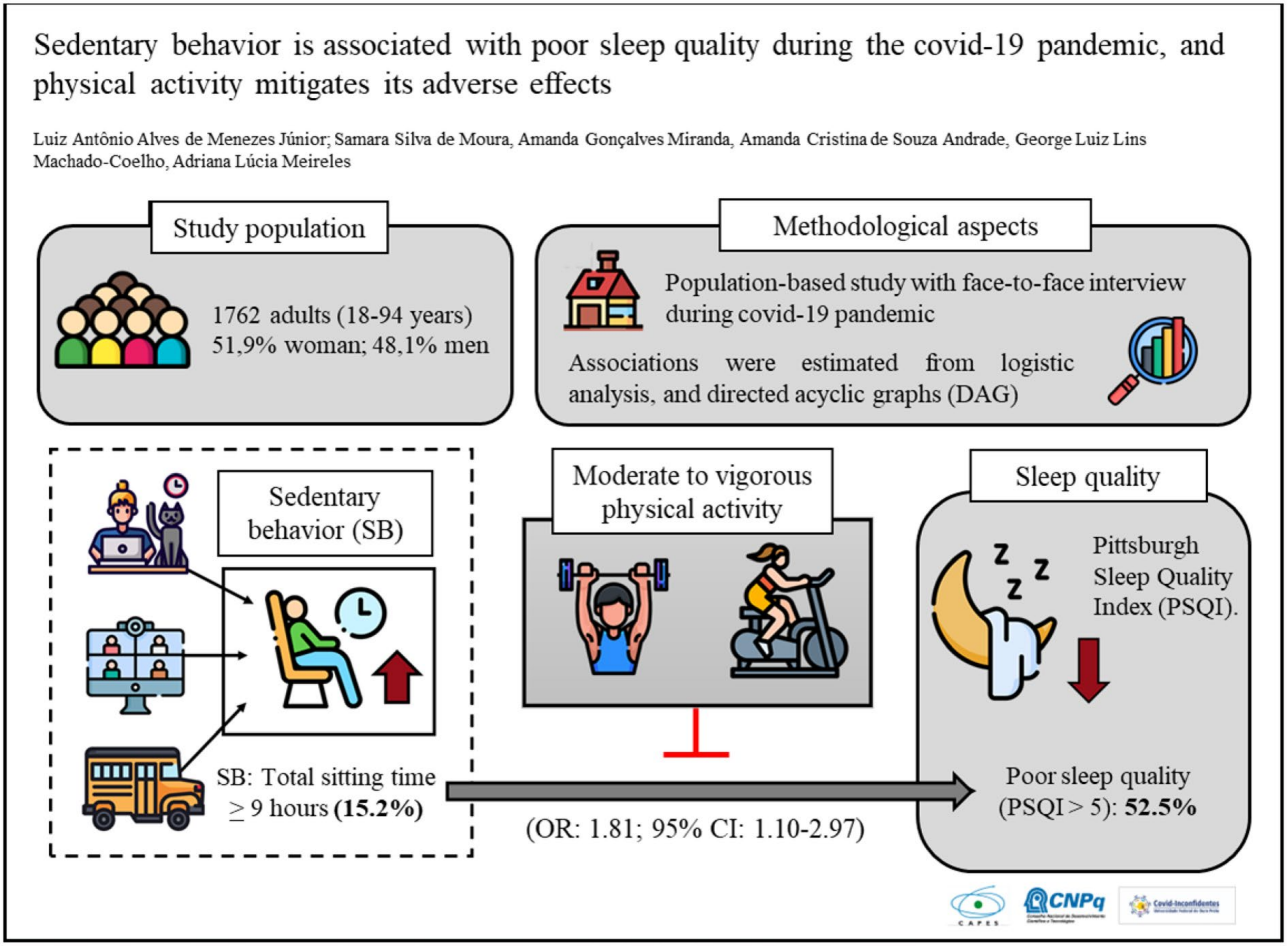

**Supplementary Information:**

The online version contains supplementary material available at 10.1186/s12889-023-16041-8.

## Introduction

During the COVID-19 pandemic, worldwide organizations promoted strategies to contain the spread of the disease, among which social restriction was highlighted [1]. Therefore, it was strongly encouraged to perform all daily activities at home, including work and leisure activities. These measures led to an increase in the time spent in sedentary behavior (SB), defined as activities with low energy expenditure [2], such as specific behaviors of sitting, reclining or lying down, reading, studying, watching television, using computers and smartphones, among others [3]. The increase in SB is concerning, given that the literature has consistently highlighted that spending excessive time on sedentary behaviors can reflect negative impairment on the health of subjects [4, 5].

Furthermore, people’s sleep quality during the pandemic was extensively affected by several pathways and mechanisms, whether direct or indirect. Directly, the effect of the virus infection on the sleep-wake cycle can be highlighted [6]. Indirectly, we can cite the fear and uncertainty related to the pandemic [7], the promotion or exacerbation of unhealthy habits and lifestyle modifications, such as increased SB [2], and reduced levels of physical activity during leisure time [8].

SB can influence important components of sleep, from its induction to its maintenance [9]. There are some hypotheses for this association, among them exposure to screens, especially at night, since screens backlit by LED can cause clinically relevant melatonin suppression and psychophysiological arousal, intervening in our biological clock [10]. Additionally, regular physical activity acts as a circadian cycle synchronizer [11] and is indispensable for mitigating the deleterious effects of SB on health [11, 12].

Recent research has sought to investigate the association between SB and sleep quality. A cross-sectional study conducted in China during the COVID-19 pandemic found that higher levels of SB were associated with poor sleep quality among college students [13]. Another cross-sectional study conducted in Japan before the pandemic found that longer screen time, a common type of SB, was associated with shorter sleep duration and lower sleep efficiency among children [14]. A longitudinal study conducted in Australia before the pandemic found that higher levels of SB were associated with an increased risk of insomnia symptoms among older adults [15]. These studies suggest that SB may have negative effects on sleep quality and other sleep parameters, which may impair health and well-being. Therefore, the association between SB and sleep quality has been evidenced in studies with specific populations, such as students, children, and the elderly. However, there is still a gap in knowledge about how this association manifests in adults. Another relevant aspect to be investigated is the role of physical activity in this relationship, since it can promote better quality sleep and can attenuate or explain the negative effects of SB on sleep.

Considering that studies associating SB with sleep quality are incipient and still poorly explored [16], this study hypothesizes that individuals with SB during the pandemic have a higher chance of poor sleep quality, and moderate to vigorous leisure-time physical activity (MVPA) may be a protective factor for sleep quality.

## Methods

### Study design

We conducted a population-based serological survey, entitled “COVID-Inconfidentes: Epidemiological surveillance of COVID-19 in the region of Inconfidentes/MG”, between October and December 2020 in two medium-sized cities in the Iron Quadrangle region of Minas Gerais (Ouro Preto and Mariana), one of the largest iron ore producing areas in Brazil.

The sample size calculation was based on the 2010 population census, considering the finite population factor, the prevalence of poor sleep quality of 50%, according to previous epidemiological surveys in adults [17–19], confidence level of 95%, design effect equal to 1.5 and precision of 5%. Based on the sample calculation, 687 and 684 individuals should have been interviewed in the cities of Ouro Preto and Mariana, respectively, totaling 1,371 individuals in the two cities evaluated. For sample selection, we adopted cluster sampling in three stages: census sector (considering the number of households and the average income of each sector); household (selected from a systematic sampling); and resident (randomly selected). Subsequently, the sample weight of each selected unit (census sector, household, and individual) was calculated and adjusted to compensate for the non-response loss of interviews and the weight of the selected household and resident.

We evaluated at first 1762 individuals, 703 from Mariana and 926 from Ouro Preto. However, we excluded 70 participants who did not complete the questionnaire on time in sedentary behavior, which was one of the main variables of interest. Therefore, our final sample was 1629 participants in the two cities. More details of the data collection are available in Meireles et al. (2021) [20].

Face-to-face interviews were conducted in the residents’ homes using an electronic form by the interviewer. The questionnaire was subdivided according to sociodemographic and economic aspects, living habits, general health conditions, and quality of sleep. All procedures were performed according to the Brazilian guidelines and standards for research involving human beings of the Declaration of Helsinki and approved by the Research Ethics Committee (Ethics Submission Certificate no. 32815620.0.1001.5149). This study followed reported guidelines dictated by the Strengthening the Reporting of Observational Studies in Epidemiology (STROBE).

### Sedentary behavior (SB)

SB was measured by total time spent sitting using the following questions: “Before the pandemic (March 2020), Monday through Friday, how much time (in hours) in total per day did you spend sitting (including time spent on mobile phone, TV, computer, tablet, books, car, public transport)?“; or “Currently (October to December 2020), Monday through Friday, how much time (in hours) in total per day did you spend sitting (include time spent on a cell phone, TV, computer, tablet, books, car, public transportation)? Therefore, this question is related to the period during the pandemic, from October to December 2020, when our data were collected.

Total sitting time in hours was classified in two forms: classification 1, every 3 h (< 3 h; 3-6 h; 6-9 h; ≥ 9 h); and classification 2, ≥ 9 h. The cut-off point for classification 2 was based on a meta-regression analysis with over 1 million individuals, suggesting that individuals with total sitting time per day greater than or equal to 9 h have higher risks for all-cause mortality [5]. We also calculated the difference between hours spent in SB during and before the pandemic, a variable we referred to as “change in sedentary behavior during the pandemic” (SB during the pandemic - SB before the pandemic).

### Physical activity in leisure time

Physical activity during leisure time was evaluated based on the VIGITEL questionnaire, which is a surveillance system of risk and protective factors for chronic diseases by telephone survey, conducted annually by the Brazilian Ministry of Health. The VIGITEL covers four domains of physical activity: leisure time, occupational activity, commuting and household activities. In this article, we evaluated only physical activity in leisure time, which was measured by the following question: “Currently, you practice any type of physical exercise or sport?” (yes or no). If the answer was ‘yes’, additional questions were asked about the type, frequency, duration and intensity of physical activities performed. This questionnaire has been validated and shown to be reliable and comparable to the Global Physical Activity Questionnaire (GPAQ), which is a reference method for measuring physical activity in different domains [21].

The intensity of physical activity was assessed according to the Compendium of Physical Activity Codes and MET Intensities, and classified as moderate physical activity: walking, treadmill walking, weight training, water aerobics, gymnastics in general, swimming, martial arts, wrestling, cycling, volleyball/foot volleyball, and dancing; and vigorous physical activities: running, treadmill running, aerobics, soccer/futsal, basketball and tennis [22, 23].

Subsequently, the ratio between time spent in moderate to vigorous physical activity (MVPA) during leisure time and time in SB was analyzed. For this, the average daily MVPA practice was calculated; weekly frequency of MVPA (0 to 7 days) x daily time of MVPA (minutes) / 7]. The time spent practicing MVPA was divided by the hours on SB according to the following equation: average daily MVPA time (minutes/day) / time on SB (hours/day). Subsequently, it was classified according to Chastin et al. (2021), who suggests the practice of at least 2.5 min of MVPA for each sedentary hour, as a way to reduce the impacts of SB [24].

### Sleep quality

Sleep quality was evaluated by the Pittsburgh Sleep Quality Index (PSQI) [25]. The Brazilian version of the PSQI had an overall reliability coefficient (Cronbach’s α) of 0.82, indicating a high degree of internal consistency [26]. This instrument is composed of 19 questions categorized into seven components, each component scoring from 0 to 3: subjective sleep quality (C1), sleep latency (C2), sleep duration (C3), habitual sleep efficiency (C4), sleep disturbances (C5), sleep medication use (C6), and daytime dysfunction (C7). The sum of the scores produces an overall score, ranging from 0 to 21, where the highest score indicates poorer sleep quality [25]. In this study, sleep quality was classified as good sleep quality when the PSQI score was less or equal than 5 (PSQI ≤ 5), and as poor sleep quality when the PSQI was greater than to 5 (PSQI > 5) [25, 26]. A moderate to severe difficulty in a sleep-specific domain (C1 to C7) was defined as a score of ≥ 2 [25].

### Covariates

The questionnaire included variables for possible confounding controls in the analysis of the association between SB and sleep quality. The variables evaluated were sex (female or male), age group (18–34 years; 35–59 years; ≥ 60 years), marital status (single or married), living status (living alone or not living alone), and self-reported skin color (white or BBYI). The self-reported skin color was based on the categories proposed by the Brazilian Institute of Geography and Statistics (IBGE), which are: White, Brown, Black, Yellow and Indigenous. BBYI is an acronym in English for Black, Brown, Yellow and Indigenous. This evaluation is valid and consistent, as Travassos et al. (2011) showed [27]. Current household income (≤ 2 minimum wages; > 2 to ≤ 4 minimum wages; > 4 minimum wages), education level (< 8 years; 9–11 years; ≥ 12 years of study), and family structure (number of residents at home and residents under 18 years of age) were also assessed. Furthermore, individuals were asked about their employment status during the pandemic, in which they were asked whether or not they were working during the pandemic (employed or unemployed), and their workplace routine, in which individuals were asked whether or not they worked from home (work from home) during the pandemic.

Health conditions included chronic diseases, chronic pain, exposure to sunlight, and body mass index (BMI), from self-reported. Chronic diseases were measured by report of medical diagnosis of the following diseases: hypertension, diabetes, asthma, lung disease, chronic kidney disease, cancer, heart disease, or thyroid disease. Individuals were combined into two categories: with morbidity (reporting at least one disease) and without morbidity (no disease). Chronic physical pain (self-reported physical pain present for 3 months or more) [28]. The average daily sun exposure was evaluated and classified as “insufficient” if exposure was < 30 min/day and “sufficient” if it was ≥ 30 min/day [29]. BMI was classified according to cut points: underweight (BMI < 18.5 kg/m² if < 60 years or BMI < 22.0 kg/m² if ≥ 60 years), eutrophic (BMI 18.5–24.9 kg/m² if < 60 years or BMI 22.0–27.0 kg/m² if ≥ 60 years), and excess weight (BMI ≥ 25.0 kg/m² if < 60 years or BMI ≥ 27.0 kg/m² if ≥ 60 years) [30, 31].

### Statistical analysis

**Fig. 1:**
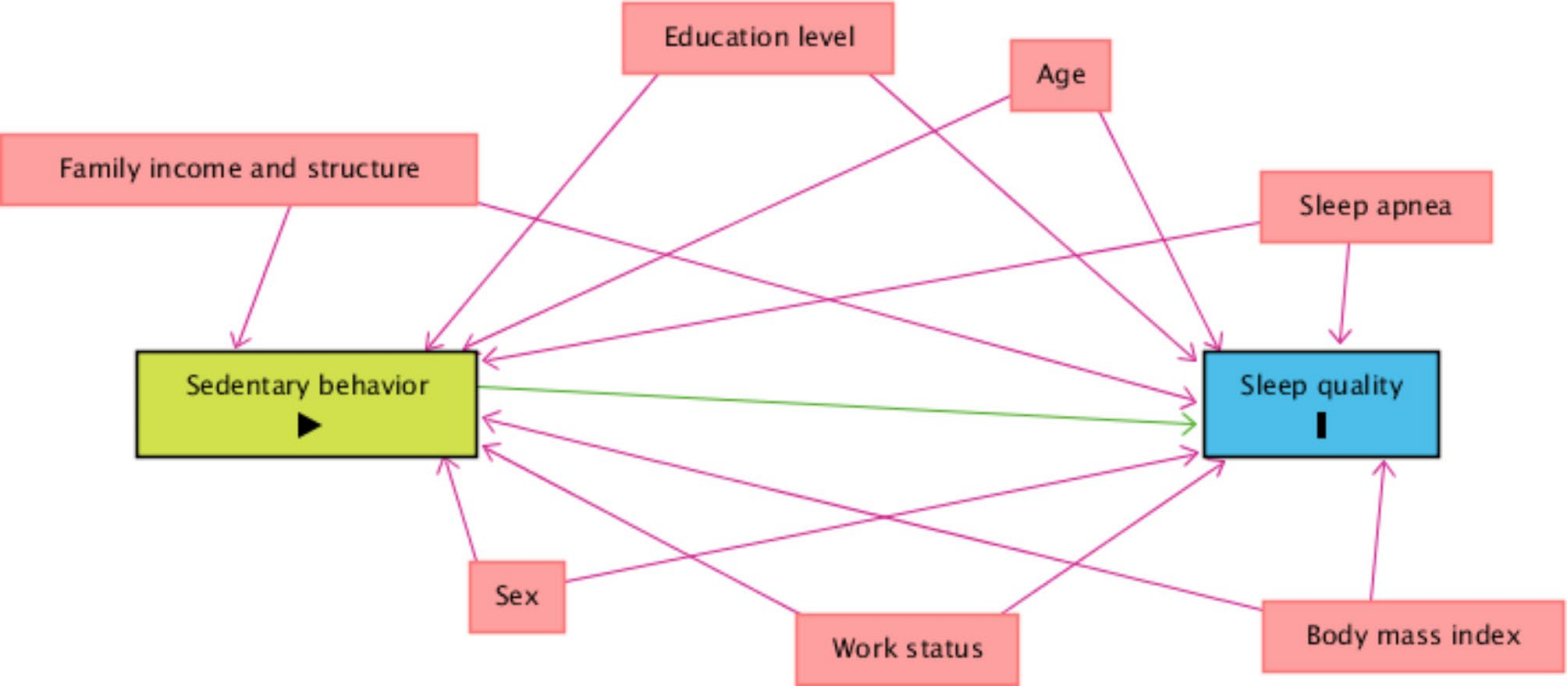
Simplified directed acyclic graph (DAG) of the association between sedentary behavior and sleep quality during the COVID-19 pandemic. Legend: The variable in green and with the “►” symbol inside the rectangle was the exposure variable; those in blue and with the letter “I” inside the rectangle were the response variables; variables in blue are the antecedents of the outcome variable; and those in red are antecedents of the outcome and exposure variables. The figure shows only the variables that were selected for multivariate modeling based on the backdoor criterion applied to the full directed acyclic graph (DAG). The variables are: age, sex, education level, family income, family structure, active worker, body mass index and medical diagnosis of sleep apnea. The arrows indicate the causal relationships between the variables. The full DAG with all the potential confounders is presented in Figure S1 of the supplementary material

A theoretical causality model based on a directed acyclic graph (DAG) was developed according to the exposure variable (SB), outcome (sleep quality), and confounding variables, using the online software Dagitty, version 3.2. Causal connections represented by arrows were established between variables (Fig. 1). Each variable in the DAG was represented by a rectangle and the colors had different meanings: green was the exposure variable; blue circled by black was the response variable; variables considered as potential confounders were included, in blue are the antecedents of the outcome variable, and those in red are the antecedents of the outcome and exposure variables. To avoid unnecessary adjustment, spurious associations, and estimation errors, the backdoor criterion was used to select a minimum set of confounding variables to fit the analyses [32]. The model was adjusted by the following minimum set of variables: sex, age, education level, household income, family structure, being an active worker, body mass index, and medical diagnosis of sleep apnea. Multicollinearity was tested by variance inflation factors (VIF < 10) [33].

Unadjusted and adjusted logistic regressions were performed for the variables indicated by DAG. In the multiple logistic models, we found that after the inclusion of the family income and education level variable, the previously non-significant odds ratio value became significant. Thus, the interactions between family income, education level, and SB of the groups studied were tested. As no significant interaction effects were found, we did not stratify the analysis by these variables, which would be recommended otherwise. Therefore, we included all individuals in the same model instead of stratifying the analysis by these variables [34]. Categorical variables were described as relative frequencies and 95% confidence interval (95%CI), and continuous variables were described as means and 95%CI. Statistical analyses were performed considering the study design and sampling weighting factors using the “svy” package of Stata® software, version 15.0. The significance level was set at 0.05.

## Results

### Characteristics of study individuals

Among the 1629 individuals evaluated, the mean time spent on SB before the pandemic was 4.6 h (95%CI: 4.3-4.9 h), and during the pandemic, it was 5.1 h (95%CI: 4.8-5.5 h). The prevalence of SB considering 9 h or more per day of total sitting time was 11.3% (95%CI: 8.6-14.8 h) before the pandemic and 15.2% (95%CI: 12.1-18.9 h) during the pandemic (Table 1).

**Table 1:** Sedentary behavior in adults before and during the covid-19 pandemic, COVID-Inconfidentes Survey (2020)

	Sedentary behavior			
	Before the pandemic	During the pandemic	Difference*	p-valor
Continues values				
Hours per day^a^	4.6 (4.3–4.9)	5.1 (4.8–5.5)	0.6 (0.3–0.8)	**< 0.001** ^**1**^
**Classification 1**				
< 3 h/day	35.2 (30.7–39.9)	39.2 (34.9–43.8)	+ 7.0%	**< 0.001** ^**2**^
3-6 h/day	29.3 (24.5–34.6)	19.4 (16.0-23.2)	-9.9%	
6-9 h/day	24.2 (19.9–29.2)	26.2 (22.1–30.6)	+ 2.0%	
≥ 9 h/day	11.3 (8.6–14.8)	15.2 (12.1–19.9)	+ 3.9%	
**Classification 2**				
< 9 h/day	88.7 (85.2–91.4)	84.8 (81.1–87.9)	-3.9%	**< 0.001** ^**2**^
≥ 9 h/day	11.3 (8.6–14.8)	15.2 (12.1–18.9)	+ 3.9%	

CI: Confidence intervals.

^*^ Change in sedentary behavior during the pandemic: Sedentary behavior during the pandemic - sedentary behavior before the pandemic.

^a^ Values are presented as mean and CI95%.

^1^ Paired t-t test was performed.

^2^ McNemar’s chi-square test was performed.

Table 2 shows the sociodemographic characteristics and health conditions of the study participants. Of the participants, 51.9% were women, the most prevalent age group was 35–59 years (45.6%), most were married (53.2%), had from 0 to 8 years of education (70.8%), and had a family income equal or less than two minimum wage (41.1%). Regarding work-related variables, 52.5% of the evaluated individuals were active workers, and 20.3% were at work from home. Concerning health conditions, 52.3% of the individuals self-reported at least one chronic disease, and 57.1% of the study population was overweight.

**Table 2:** Sociodemographic, health, and lifestyle characteristics during the pandemic according to sedentary behavior, COVID-Inconfidentes Survey (2020)

		Sedentary behavior			
Characteristics	Total % (CI95%)	< 9 h	≥ 9 h	OR	p
Sex					
Male	48.1 (41.0-55.2)	48.8 (40.6–57.0)	47.4 (37.8–58.4)		
Female	51.9 (44.7–59.0)	51.2 (43.0-59.4)	52.6 (41.6–63.2)	1.06 (0.64–1.73)	0.828
**Age**					
18–34 years	35.6 (31.1–40.3)	32.9 (27.8–38.4)	56.2 (45.7–66.1)	1.00	
35–59 years	45.6 (41.1–50.2)	46.9 (42.0-51.9)	35.7 (26.7–45.9)	0.44 (0.28–0.71)	**0.001**
≥ 60 years	18.8 (15.5–22.7)	20.2 (16.1–24.9)	8.1 (4.5–14.2)	0.24 (0.11–0.49)	**< 0.001**
**Skin color** ^1^					
White	25.6 (20.8–31.2)	23.5 (18.2–29.9)	43.1 (31.9–55.0)	1.00	
BBYI	74.4 (68.8–79.2)	76.5 (70.1–81.8)	56.9 (45.0-68.1)	0.41 (0.22–0.73)	**0.002**
**Marital status** ^2^					
Married	53.2 (47.2–59.2)	56.5 (49.6–63.2)	32.4 (23.0-43.5)	1.00	
Not married	46.8 (40.8–52.8)	43.5 (36.8–50.4)	67.6 (56.5–77.0)	2.71 (1.56–4.73)	**0.001**
**Education level** ^3^					
0–8 years	70.8 (65.8–75.3)	67.3 (62.1–72.0)	90.4 (84.0-94.4)	1.00	
9–11 years	27.5 (23.0-32.5)	30.8 (26.0-36.1)	9.2 (5.3–15.6)	2.50 (1.21–5.18)	**0.014**
≥ 12 years	1.7 (7.6–3.7)	1.9 (0.9–4.3)	0.4 (0.1–0.6)	8.03 (4.31–14.97)	**< 0.001**
**Family income** ^4^					
≤ 2 MW	41.1 (35.6–46.8)	47.9 (42.1–53.7)	26.1 (18.8–34.9)	1.00	
> 2 to ≤ 4 MW	32.0 (26.9–37.5)	30.0 (24.6–36.0)	31.9 (22.6–43.0)	1.96 (1.11–3.43)	**0.020**
> 4 MW	26.9 (22.0-32.5)	22.1 (17.3–27.8)	42.0 (30.8–54.1)	3.49 (2.00-6.09)	**< 0.001**
**Family structure** ^5^					
No residents aged under 18 years old	49.4 (44.6–54.3)	47.1 (42.1–52.2)	62.6 (51.9–72.1)	1.00	
≥ 1 residents aged under 18 years old	50.6 (45.7–55.4)	52.9 (47.8–57.9)	37.4 (27.9–48.1)	0.53 (0.33–0.85)	**0.008**
**Work status** ^6^					
Not workers	45.4 (40.2–50.6)	46.3 (41.2–51.4)	40.4 (30.1–51.6)	1.00	
Active workers	54.6 (49.4–59.8)	53.7 (48.6–58.8)	59.6 (48.4–69.9)	1.27 (0.83–1.93)	0.262
**Work from home** ^7^					
No	79.7 (75.0-73.7)	80.9 (75.6–85.2)	67.0 (57.1–75.6)		
Yes	20.3 (16.3–25.0)	19.1 (14.8–24.4)	33.0 (24.4–42.9)	2.08 (1.29–3.36)	**0.003**
**Nutritional status** ^8^					
Eutrophic	40.3 (34.7–47.5)	41.7 (34.6–49.2)	42.0 (30.0–55.0)	1.00	
Underweight	2.6 (2.0-4.1)	3.2 (2.2–4.6)	2.0 (0.8–5.1)	0.62 (0.20–1.87)	0.388
Excess weight	57.1 (29.5–44.9)	57.3 (50.0-64.3)	56.0 (43.4–67.9)	0.92 (0.52–1.65)	0.789
**Chronic diseases** ^9^					
No	47.7 (41.3–54.2)	48.5 (41.1–56.0)	48.5 (36.5–60.8)	1.00	
Yes	52.3 (45.8–58.7)	51.5 (44.1–58.9)	51.5 (39.2–63.5)	0.99 (0.57–1.74)	0.993
**Chronic pain** ^10^					
No	65.7 (61.4–69.7)	65.7 (60.6–70.5)	68.0 (57.8–76.7)	1.00	
Yes	34.3 (30.3–38.6)	34.3 (29.5–39.4)	32.0 (23.3–42.2)	0.90 (0.54–1.50)	0.686
**Sleep apnea** ^11^					
No	93.6 (91.4–95.2)	94.2 (91.9–95.9)	89.8 (80.9–94.8)	1.00	
Yes	6.4 (4.8–8.6)	5.8 (41 − 8.1)	10.2 (5.2–19.1)	1.85 (0.79–4.32)	0.146
**Exposure sun** ^12^					
≥ 30 min/day	65.6 (59.5–71.3)	67.7 (60.7–73.9)	54.4 (43.2–65.3)	1.00	
< 30 min/day	34.4 (28.7–40.5)	32.3 (26.1–39.3)	45.6 (34.7–56.8)	1.75 (1.02-3.00)	**0.040**

CI = confidence interval; OR = odds ratio; MW = minimum wage.

^1^ BBYI = Brown, black, yellow and indigenous;

^2^ Not married: Widowed, divorced, or single;

^3^ Education level: years of schooling;

^4^ Minimum wage of the year in which data collection occurred, 2020 - BRL R$:1045,00 ≈ USD 194.25 (1 USD = 5.3797 BRL);

^5^ Family structure: Number of residents younger than 18 years old in the household;

^6^ Not workers: Unemployed, pensioner, or retiree;

^7^ Active workers who were working at home;

^8^ Underweight (BMI < 18.5 kg/m² if < 60 years or BMI < 22.0 kg/m² if > 60 years), eutrophic (BMI 18.5–24.9 kg/m² if < 60 years or BMI 22.0-27.9 kg/m² if > 60 years), excessweight (BMI ≥ 25.0 kg/m² if < 60 years or BMI ≥ 28.0 kg/m² if > 60 years);

^9^ Chronic diseases, measured by report of medical diagnosis of the following diseases: hypertension, diabetes, asthma, lung disease, chronic kidney disease, cancer, heart disease, or thyroid disease. Individuals were combined into two categories: with morbidity (reporting at least one disease) and without morbidity (no disease);

^10^ Chronic physical pain (self-reported physical pain present for 3 months or more);

^11^ Sleep apnea measured by report of medical diagnosis;

^12^ The average daily sun exposure was evaluated.

### Sedentary behavior and sleep quality

To evaluate the association of SB during the pandemic with sleep quality, we verified that in classification 1 (< 3 h; 3-6 h; 6-9 h; ≥ 9 h), the chance of poor sleep quality was higher only for individuals with SB of 9 h or more (OR: 1.74; 95%CI: 1.02–2.97) compared to individuals with less than 3 h of SB. For classification 2 (< 9 h and ≥ 9 h), the chance of poor sleep quality was 77% higher in subjects with SB of 9 h or more (OR: 1.77; 95%CI: 1.06–2.95) compared with individuals with less than 9 h of SB. Furthermore, each increase of 1 h in SB increased by 8% the chance of poor sleep quality (OR: 1.08; 95%CI 1.01–1.15) (Table 3).

**Table 3:** Association of sedentary behavior (SB) during the pandemic with sleep quality, COVID-Inconfidentes Survey (2020)

Sedentary behavior		Unadjusted analysis		Adjusted analysis*	
		OR (CI95%)	p	OR (CI95%)	p
**Classification 1**	< 3 h	1.00		1.00	
	3-6 h	0.86 (0.53–1.39)	0.533	0.94 (0.59–1.48)	0.774
	6-9 h	0.81 (0.37–1.74)	0.583	1.00 (0.56–1.79)	0.998
	≥ 9 h	1.08 (0.55–2.11)	0.818	**1.74 (1.02–2.97)**	**0.043**
**Classification 2**	< 9 h	1.00		1.00	
	≥ 9 h	1.20 (0.71–2.02)	0.501	**1.77 (1.06–2.95)**	**0.031**
**Change in sedentary behavior** ^1^	+ 1 h/day	1.05 (0.97–1.14)	0.197	**1.08 (1.01–1.15)**	**0.049**

p-values: Wald’s test was used to assess the significance of ORs. Bold values statistical significance at 5% alpha level.

* Multivariate logistic regression adjusted according to directed acyclic graph. Adjusted for age, sex, education level, family income, family structure, active worker, body mass index and medical diagnosis of sleep apnea.

^1^ Change in sedentary behavior: The difference between hours spent in sedentary behavior (SB) during and before the pandemic was calculated, according to a following equation: [SB during the pandemic (hours/day) - SB before the pandemic (hours/day)].

In addition, we also explored the association of SB with each component of the PSQI. The results showed in a multivariate analysis that SB had a significant association with subjective sleep quality (OR:1.47;95%CI:1.05–2.05), sleep latency (OR:1.53;95%CI:1.15–2.04), sleep disturbances (OR:1.48;95%CI:1.09-2.00), use of sleeping medication (OR:1.90;95%CI:1.25–2.88), and daytime dysfunction (OR:1.49;95%CI:1.01–2.18) (Figure S2).

The ratio MVPA/SB was lower among individuals with SB ≥ 9 h per day than among those with SB < 9 h per day (mean: 1.50 min; 95% CI: 0.95–2.05 vs. mean: 5.87 min; 95% CI: 4.64–7.10; p < 0.001). In multivariate analysis, the ratio analysis of MVPA per SB verified that in individuals with SB ≥ 9, the practice of 1 min of MVPA per hour of SB decreased by 19.0% the chance of poor sleep quality (OR: 0.84; 95%CI: 0.73–0.98). Evaluating the cutoff suggested in the literature, the chance of poor sleep quality was 3 times lower in individuals who performed 2.5 min or more of MVPA per hour of SB compared to individuals who performed less than 2.5 min of MVPA per hour of SB (OR: 0.33; 95%CI: 0.16–0.72) (Table 4).

**Table 4:** Association between sleep quality and moderate to vigorous leisure-time physical activity for each hour in sedentary behavior, according to the sedentary behavior classification, COVID-Inconfidentes Survey (2020)

	Sedentary behavior			
	< 9 h/day (84.8%)		≥ 9 h/day (15.2%)	
MVPA minutes per hour of SB				
(CI95%)	5.87 (4.64–7.10)		1.50 (0.95–2.05)	
Association with poor sleep quality*	**OR (CI95%)**		**OR (CI95%)**	
**Continues value**				
1 min of MVPA per hour of SB	1.00 (0.99–1.01)		**0.84 (0.73–0.98)**	
**Classification**				
< 2,5 min of MVPA per hour of SB	1.00		1.00	
≥ 2,5 min of MVPA per hour of SB	0.86 (0.54–1.37)		**0.33 (0.16–0.72)**	

MVPA: Moderate to vigorous leisure-time physical activity. SB: Sedentary behavior. OR: Odds ratio; CI: Confidence interval.

The ratio MVPA/SB was calculated by dividing the minutes of moderate to vigorous leisure-time physical activity (MVPA) per day by the hours of sedentary behavior (SB) per day. The values presented are the mean and 95% confidence interval (CI) of the ratio MVPA/SB for each category of SB.

*Multivariate logistic regression adjusted according to directed acyclic graph. Adjusted for age, sex, education level, income, family structure, active worker and body mass index.

Values in bold indicates statistical significance (p-value < 0.05).

## Discussion

These findings corroborate initial hypotheses that adult individuals with SB have higher chances of poor sleep quality during the pandemic. Furthermore, in individuals with SB, the practice of MVPA is associated with fewer chances of poor sleep quality. Our findings may contribute to the development of further research aimed at defining the effects of SB on human health, and its relationship with sleep quality, especially during the COVID-19 pandemic.

SB has increasingly been recognized as a serious public health problem and recommendations have begun to appear in public health guidelines [35] suggesting that all adults must reduce the amount of SB daily. Notably, SB has been consistently characterized as a major risk factor for several chronic diseases and all-cause mortality [4]. SB is estimated to be responsible for 3.8% of all-cause mortality in adults, independently of the level of physical activity, according to a meta-analysis of 54 countries [36].

The COVID-19 pandemic has directly affected some health behaviors, such as the increase in SB. As evidenced by Wang et al. (2020), in a study of 2289 Chinese adults, between March and April 2020, in which an increase of more than 60% in time SB was observed [37]. A similar was observed in Brazil, in a study of 39,693 adults, where an increase of up to 266% in SB was observed during the pandemic, accompanied by an increase in physical inactivity [38]. In our study, the prevalence of high SB increased by 34.5%. These results suggest that the impact of the pandemic on SB may vary according to the context and characteristics of the population. For example, the Brazilian study included a larger and more diverse sample than ours, which may explain the higher increase in SB observed there. Moreover, the Brazilian study used a different cutoff point for high SB (4 h per day) than ours (9 h per day), which may reflect different levels of risk associated with SB in different populations.

The relationship between SB and sleep is still recent in research, and studies mostly evaluated only some components of sleep [39]. As shown by Yang et al. (2017) in a systematic review of 16 studies, in which SB was associated with an increased risk of insomnia and sleep disorders. However, they found no association with overall poor sleep quality [16]. Jeong et al. (2021), evaluating 224,118 South Koreans, found the chance of poor sleep quality was 12 to 26% higher in individuals with sedentary time over 4 h/day [40]. Therefore, our results are relevant, the chance of poor sleep quality was higher in individuals with SB of 9 h or more per day, or who increased this behavior during the pandemic.

One possible mechanism by which sedentary behavior may impair sleep quality is by altering the environmental and social circadian rhythm synchronizing agents (zeitgebers), such as natural and artificial light exposure, media use, and social interactions. These factors can affect the production of melatonin, a hormone that induces sleepiness at night. Sedentary behavior may reduce exposure to natural light during the day, which is essential for aligning the circadian rhythm with the day-night cycle and regulating melatonin secretion. On the other hand, sedentary behavior may increase exposure to artificial light and electronic stimulation at night, which can inhibit melatonin production and stimulate the brain in different ways that impair relaxation and sleep onset [41]. Regardless of checking notifications on the phone, following social media, attending meetings via video call, watching television, or spending extra hours staring at a computer while working, SB is associated with a large amount of time spent in front of backlit screens [42]. Excessive exposure time to backlit screens can interfere with circadian rhythm alignment, and stimulate the brain in different ways that impair relaxation, a crucial point for good sleep quality [43, 44]. This stage of sleep can be extensively affected by excessive information from electronic devices, social and journalistic media, gaming, or messaging and emails that require a certain level of cognitive alertness. These and other interactive activities on electronic devices can be potentially overstimulating, leading to a drawback in circadian rhythms and increasing sleep latency [45]. The same is observed for passive technology, such as a television in the background or a cell phone that emits sounds, vibrations, and light, which can also affect alertness levels and the production of melatonin, affecting sleep quality [45]. This result was corroborated by Kakinami et al. (2017) in a study of Canadian adults, in which they found that each additional hour of TV and computer use per day was associated with a 17% and 13% increase, respectively, in the odds of reporting poor sleep quality [9].

Another clinically relevant effect of electronic devices on sleep quality is blue light [43]. Being a part of the visible light spectrum with shorter wavelengths than other colors in the visible light spectrum causes more alertness than warmer shades of light. This type of light has great effects on alertness, the drawback of circadian rhythm, hormone production, and sleep cycles [46]. This wavelength of light is emitted by LED and fluorescent lamps, as well as many electronic devices. While the light of any type can suppress melatonin secretion, blue light, especially at night, does more intensely [47]. As demonstrated by Lockley et al. (2003) in an experiment comparing the effects of 6.5 h of blue light exposure with exposure to other lights of comparable brightness. The blue light suppressed melatonin and disrupted circadian rhythms about two times more [47]. This data is concerning, given that a recent study by the National Sleep Foundation, found that about 80% of American individuals report looking at screens frequently during the day, 68% at night, and 58% reported looking at screens one hour before bedtime [48].

Furthermore, the pandemic potentially worsened this scenario, because, in the attempt to follow the news about the pandemic, individuals could increase their exposure time to screens, and increase their stress and anxiety related to pandemic news, generating an additive effect for poor sleep quality [42]. Thus, excessive or inadequately timed exposure to artificial light can cause a disrupted circadian rhythm to become misaligned with a person’s day-night schedule.

Besides the factors related to the circadian cycle, other possible mechanisms justify the association of SB with sleep. One of them is the metabolic, hormonal, inflammatory, and psychological changes that sedentary behavior causes and that can directly affect sleep [49]. For instance, SB can reduce energy expenditure and increase the risk of obesity, diabetes, and cardiovascular diseases [50]. These conditions can impair sleep quality by causing physical discomfort, pain or breathing problems. Furthermore, SB can interfere with the regulation of stress hormones such as cortisol [51]. This hormone can affect the circadian cycle and sleep induction by altering the balance between arousal and relaxation. Another possible mechanism is the pro-inflammatory effect of SB [50, 51]. It can disturb the immune system and increase the risk of insomnia and obstructive sleep apnea. These sleep disorders can affect the quality and quantity of sleep by disrupting the normal sleep stages and cycles. Finally, SB can associate with negative psychological factors, such as depression, anxiety, and low self-esteem [51]. These factors can compromise sleep quality by increasing negative thoughts, worries, and emotions that interfere with relaxation and sleep onset.

Another theoretical model is the homeostatic dysregulation model, which proposes that SB can affect sleep quality through two mechanisms: dysregulation of sleep pressure and dysregulation of body temperature [52]. According to this model, SB can reduce sleep pressure, which is the physiological need to sleep that accumulates throughout the day, as SB involves a low energy expenditure and low brain activation. As a result, SB can make it harder to fall asleep and stay asleep. Furthermore, SB can dysregulate body temperature, which is an important signal for sleep induction and maintenance, as SB prevents the natural drop in body temperature that occurs at night. This drop is essential for initiating and sustaining deep sleep stages.

We can verify some of these hypotheses in the analysis of the relationship of sedentary behavior with each component of the PSQI. The PSQI has seven components: subjective sleep quality, sleep latency, sleep duration, habitual sleep efficiency, sleep disturbances, use of sleep medications, and daytime dysfunction. We found that SB is associated with five of these components. These results suggest some possible mechanisms by which sedentary behavior may affect sleep quality. First, sedentary behavior can worsen the subjective perception of sleep. This can happen because sedentary behavior reduces energy expenditure and physical fatigue [50]. These factors can facilitate sleep onset and maintenance. Moreover, sedentary behavior can associate with increased psychological and emotional stress [51]. This stress can interfere with subjective sleep quality [49]. Second, sedentary behavior can increase sleep latency. This can happen because sedentary behavior decreases exposure to natural light and alters circadian rhythms [53]. These rhythms are responsible for regulating the sleep-wake cycle. In addition, sedentary behavior can increase exposure to artificial light and electronic stimulation [53]. These stimuli can inhibit melatonin production and make sleep onset more difficult [16, 49]. Third, sedentary behavior can reduce sleep efficiency. This can happen because sedentary behavior impairs cardiovascular and metabolic health [54]. These factors can influence sleep quality and continuity [49]. Fourth, sedentary behavior can increase the use of sleep medications. This can happen because sedentary behavior aggravates insomnia and sleep quality problems, leading people to turn to medications as a way to relieve symptoms. However, the use of sleep medications can have adverse effects, such as dependence, tolerance, daytime sleepiness, and cognitive changes [55]. And finally, sedentary behavior can impair daytime functioning. This can happen because sedentary behavior compromises the quality and quantity of sleep, negatively affecting mood, attention, memory, concentration, and productivity during the day. Furthermore, sedentary behavior can reduce physical activity and social interactions [51]. These factors can improve well-being and daytime functionality.

The emergence of research like this provides guidelines for clinical instructions for the general population. Recent data in the literature suggest that SB is an important factor to be evaluated in individuals with poor sleep quality. However, SB in some individuals can be caused by characteristics intrinsic to their routines, such as the way they study, work, and the means of locomotion. Some strategies can be used to mitigate its damage to health, such as including the practice of physical activity, and an important zeitgeber (environmental zeitgebers such as sunlight and social interactions, all of which contribute to regulating the circadian rhythm). Furthermore, physical activity can improve metabolism, cardiovascular health, hormonal regulation, inflammatory response, and psychological well-being, contributing to more restorative sleep [56]. In this regard, we found that practicing one minute of MVPA, per hour of SB, is associated with fewer chances of poor sleep quality in individuals with SB for 9 h or more per day. This result is supported by a recent study by Chastin et al. (2021), in which they evaluated dose-response associations between the balance of time spent in physical activity and SB with all-cause mortality. Using data from the 2005–2006 National Health and Nutrition Examination Survey, obtained by accelerometers, they found that engaging in 2.5 min or more of MVPA per hour of daily SB was associated with the same magnitude of all-cause mortality risk reduction obtained by being physically active according to current WHO recommendations [24]. Therefore, we find that this result also applies to sleep quality, and can be used to provide evidence, interventions, and recommendations for SB and health outcomes. Moreover, poor sleep quality can potentially be a mediating factor between high SB and increased mortality rates. Since SB is associated with poor sleep quality, sleep is an important risk factor for all-cause mortality [57].

Our study has some strengths. To our knowledge, this is the first study to evaluate the association of SB with sleep and the ratio of MVPA to SB on sleep quality during the COVID-19 pandemic in Brazil. In addition, the sample design brings robustness to the study: (i) a representative random sample of the resident population from different socioeconomic strata; (ii) assessment by household survey; (iii) a face-to-face study during the COVID-19 pandemic. Furthermore, the DAG was used to guide analysis plans and decisions about possible confounders. Finally, it is worth mentioning that we conducted our study in two Brazilian cities, which have a miscegenated population, and there is a lack of studies on the subject specifically in the Brazilian population. This may influence the genetic and environmental factors that affect SB and sleep quality, such as circadian rhythms, melatonin production, and light exposure. Some of the studies were conducted in populations with a predominant ethnicity, such as Asian [13, 14] or Caucasian [58], which may limit the applicability of their findings to other ethnic groups. Therefore, our study provides valuable information on the association between SB and sleep quality in a diverse and heterogeneous population, which may increase the external validity and generalizability of the results.

However, although our findings provide relevant insights, this study has limitations in some areas that deserve to be mentioned. First, the information obtained is self-reported, so the individual’s perception may overestimate and/or underestimate compared to objective measures. For example, an accelerometer could have been used to measure the individuals’ SB and sleep parameters. Physical activity was evaluated only in the leisure time domain and may lead to an underestimation of total physical activity, especially for certain population groups who may perform activities in other domains more frequently. While objective measures of sleep quality are important, we were unable to perform them due to limitations imposed by the pandemic. Our study was conducted during the second wave of the pandemic when many new cases were emerging and there was no access to vaccines in Brazil, which compromised the safety of researchers and participants. Therefore, we opted to perform only subjective evaluations to minimize the risk of contagion. We recognize that subjective evaluations may have limitations, but we believe that they still provide valuable information about individuals’ perceptions of sleep quality, especially in a pandemic context where objective measures may not be feasible. Self-report measures of SB have an acceptable level of reliability [59]. Another limitation is the inability to infer causality between SB and sleep quality due to the cross-sectional design of the study. Although we adjusted our models for several potential confounders based on the counterfactual DAG approach, we cannot rule out the presence of residual or unmeasured factors that may affect the association. Furthermore, we cannot rule out the possibility of reverse causality, i.e., that poor sleep quality may increase SB rather than the other way around. We performed a sensitivity analysis including only individuals who were not sedentary before the pandemic and found no evidence of reverse causality in this direction. However, this analysis is still limited by the cross-sectional design and the self-reported measure of pre-pandemic SB. Longitudinal studies are needed to confirm our findings and clarify the direction of the association. Furthermore, we did not assess all possible chronic sleep disorders. Although we assessed the previous presence of obstructive sleep apnea, one of the most prevalent chronic sleep disorders in Brazil [60], we did not consider other sleep disorders, such as chronic insomnia, for example.

These results could provide insights that guide relevant public health implications, lead interventions that cause changes in behaviors to benefit health, and inform physicians and health professionals about best practices based on the available evidence. The positive association between SB and poor sleep quality may increase the chances of adverse health events. Therefore, our results indicate attention and reinforce the importance of evaluating and monitoring the SB of the population, especially during social restrictions, such as during the COVID-19 pandemic. Therefore, highlighting the importance of health promotion guidelines from public agents, and multidisciplinary interventions encouraging the reduction of SB for better sleep quality.

## Conclusion

Most of the study individuals had poor sleep quality, and SB during the pandemic was a factor associated with poor sleep quality. In addition, the practice of MVPA in individuals with SB is associated with fewer chances of poor sleep quality. The results of this study may contribute to the development of further studies evaluating the causality between SB and sleep quality.

## Electronic supplementary material

Below is the link to the electronic supplementary material.

Supplementary Material 1

Supplementary Material 2

## Data Availability

The datasets generated and/or analyzed as part of the current study are not publicly available due to confidentiality agreements with subjects. However, they can be made available solely for the purpose of review and not for the purpose of publication from the corresponding author upon reasonable request.
