# Supplementary material for: Sedentary behavior is associated with poor sleep quality during the COVID-19 pandemic, and physical activity mitigates its adverse effects
Source: BMC Public Health. 2023 Jun 12;23:1116. doi: 10.1186/s12889-023-16041-8 (PMC10258778; doi:10.1186/s12889-023-16041-8)
Supplement: Supplementary file 1 — Supplementary Material 1 [file 12889_2023_16041_MOESM1_ESM.docx]

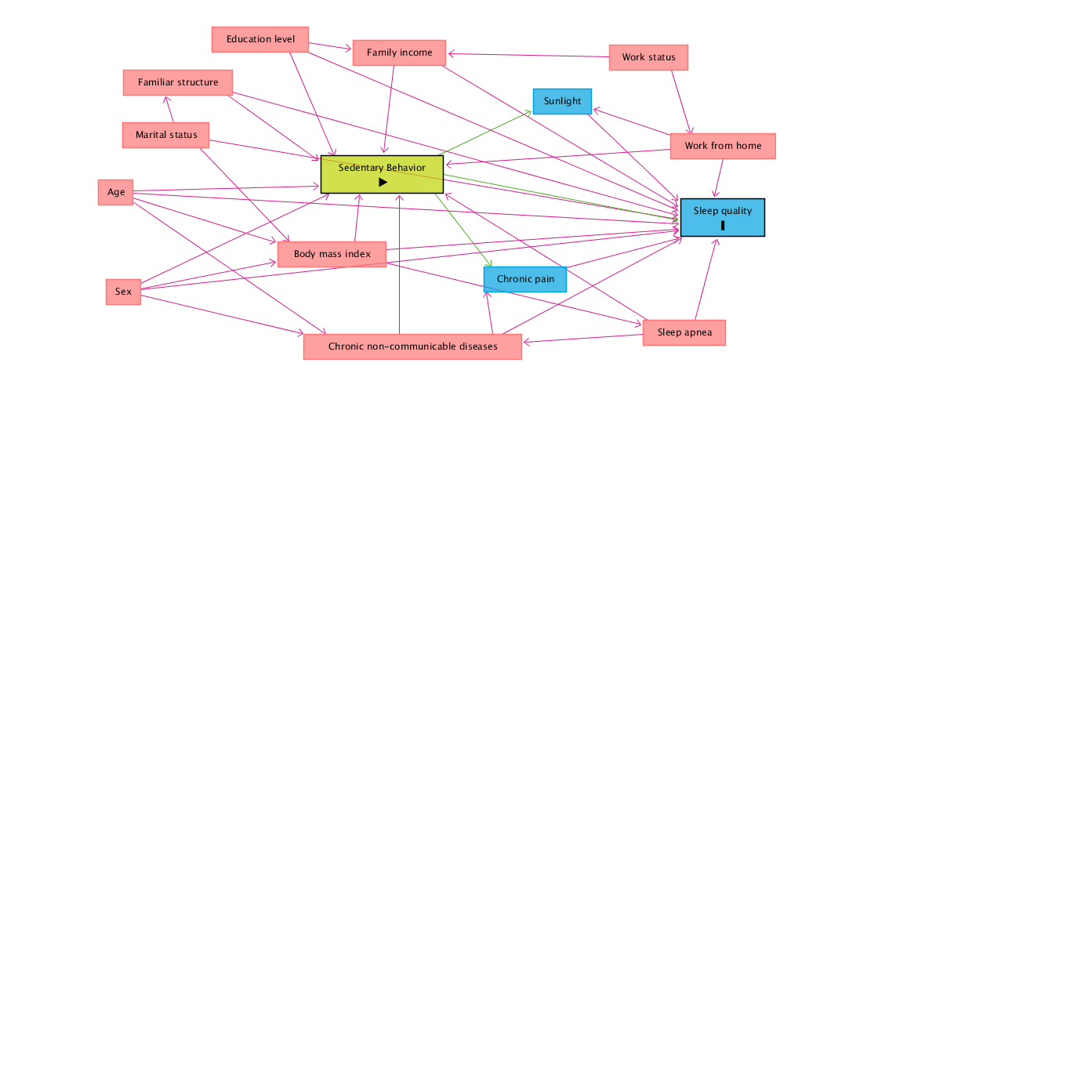


**Supplementary figure 1:** Directed acyclic graph (DAG) on sedentary behavior and sleep quality in adults during the covid-19 pandemic.

**Legend:** The variable in green and with the “►” symbol inside the rectangle was the exposure variable; those in blue and with the letter “I” inside the rectangle were the response variables; variables in blue are the antecedents of the outcome variable; and those in red are antecedents of the outcome and exposure variables.
