# Supplementary material for: Sedentary behavior is associated with poor sleep quality during the COVID-19 pandemic, and physical activity mitigates its adverse effects
Source: BMC Public Health. 2023 Jun 12;23:1116. doi: 10.1186/s12889-023-16041-8 (PMC10258778; doi:10.1186/s12889-023-16041-8)
Supplement: Supplementary file 2 — Supplementary Material 2 [file 12889_2023_16041_MOESM2_ESM.docx]

**Supplementary figure 2.** Association of sedentary behavior with moderate to difficulties Pittsburgh Sleep Quality Index subdomains.

**
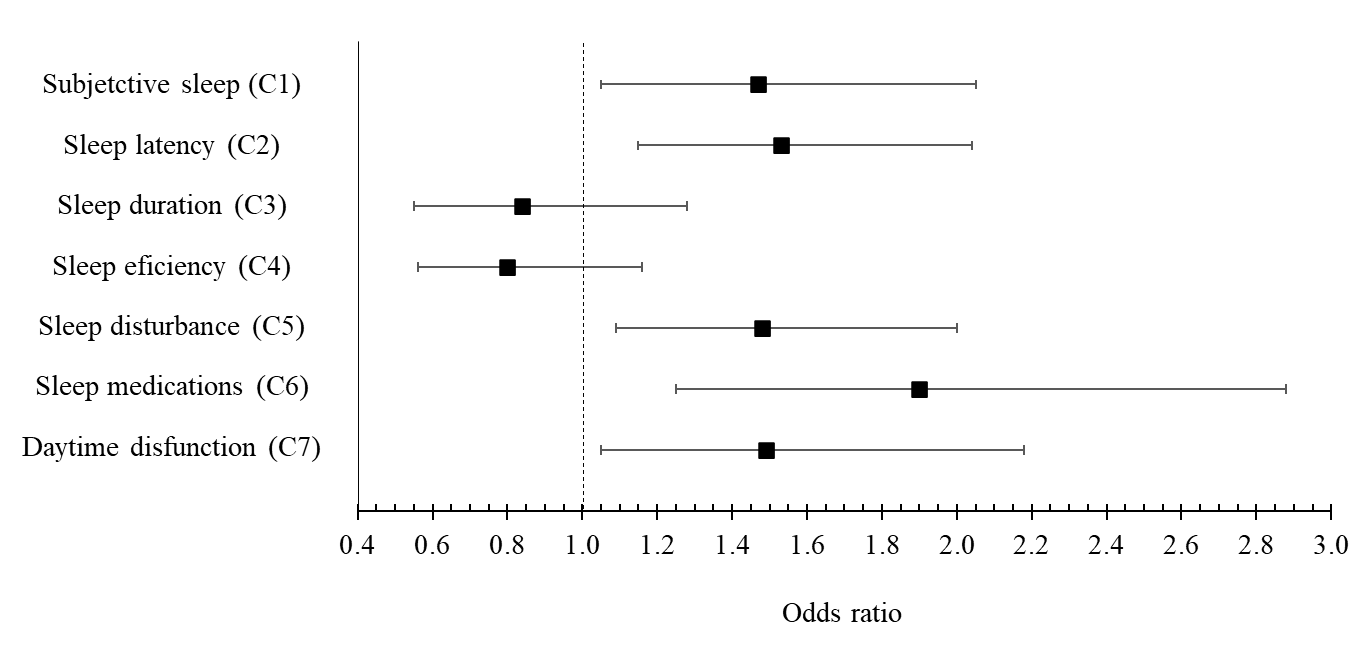
**

**Subtitle:** Performed multivariate logistic regression adjusted according to directed acyclic graph. Adjusted for age, sex, education level, family income, family structure, active worker, body mass index and medical diagnosis of sleep apnea. The outcome variable was moderate to difficulty in the sleep domain and exposure variable was sedentary behavior (> 9h/day). The score for each domain ranges from 0 to 3 (no difficulty to severe difficulty), and a domain score ≥ 2 indicates moderate to difficulty in the sleep domain.
